# Novel Imaging Technologies for Accurate Assessment of Cardiac Allograft Performance

**DOI:** 10.1007/s40472-023-00400-w

**Published:** 2023-07-17

**Authors:** Manuela Lopera Higuita, Rohil Jain, Asishana A. Osho, S. Alireza Rabi, Timothy L. Pruett, Richard N. Pierson, Paul A. Iaizzo, Shannon N. Tessier

**Affiliations:** 1 Center for Engineering in Medicine and Surgery, Massachusetts General Hospital and Harvard Medical School, Boston, MA, USA; 2 Shriners Hospitals for Children, Boston, MA, USA; 3 Division of Cardiac Surgery, Corrigan Minehan Heart Center, Massachusetts General Hospital, MA, Boston, USA; 4 Division of Solid Organ Transplantation and Department of Surgery, University of Minnesota, Minneapolis, MN, USA; 5 Department of Surgery and Center for Transplantation Sciences, Massachusetts General Hospital and Harvard Medical School, Boston, MA, USA; 6 Visible Heart Laboratories, Departments of Surgery and Biomedical EngineeringInstitute for Engineering in Medicine, University of Minnesota, MN 55455 Minneapolis, USA

**Keywords:** Heart transplant, Heart assessment, Imaging techniques, Ultrasound, Magnetic resonance imaging, Spectroscopy

## Abstract

**Purpose of the Review:**

The current lack of objective and quantitative assessment techniques to determine cardiac graft relative viability results in risk-averse decision-making, which negatively impact the utilization of cardiac grafts. The purpose of this review is to highlight the current deficiencies in cardiac allograft assessment before focusing on novel cardiac assessment techniques that exploit conventional and emerging imaging modalities, including ultrasound, magnetic resonance, and spectroscopy.

**Recent Findings:**

Extensive work is ongoing by the scientific community to identify improved objective metrics and tools for cardiac graft assessment, with the goal to safely increasing the number and proportion of hearts accepted for transplantation.

**Summary:**

This review briefly discusses the in situ and ex vivo tools currently available for clinical organ assessment, before focusing on the individual capabilities of ultrasound, magnetic resonance, and spectroscopy to provide insightful, non-invasive information regarding cardiac graft functional and metabolic status that may be used to predict outcome after transplantation.

## Introduction

Heart transplantation is the standard of care for end-stage heart diseases as it increases survival rate of affected individuals [1]. However, fewer transplants are performed every year relative to the incidence of advanced heart failure, leading to a 30% increase of patients on the heart transplant waiting list between 2009 and 2020 in the USA alone. Paradoxically, the severe organ shortage comes at a time when the number of available donors has substantially increased, suggesting utilization of available grafts is probably suboptimal [2]. One explanation for the apparent underutilization of cardiac grafts is dependence on subjective, historically based selection criteria that lack a robust evidence base. Associations between clinical parameters (prolonged cold ischemic time, increased donor age, gender mismatch) and adverse outcomes in registry database analyses have resulted in adoption of stringent donor selection standards by transplant centers. As such, only one in every three hearts is accepted for transplantation from donors where at least one other organ is procured. The current practices for donor heart utilization lead to the under-utilization of potentially viable hearts, resulting in over 40% of patients on the heart transplant waiting list to either die or to be removed from the list after becoming ineligible for transplantation due to disease progression [3].

The most promising way to safely expand the utilization of cardiac grafts is via the resuscitation and assessment of “marginal” or extended criteria organs using machine perfusion technologies, such as the organ care system (OCS), currently approved by the FDA and widely utilized around the world. Although this technology does overcome some limitations of static cold storage (e.g., uncontrolled, variable storage temperatures or inability to assess metabolic status of the allograft in real time ex vivo), the current assessment techniques lack objective and quantitative parameters to accurately predict transplantation outcomes, posing a significant impediment for the use of extended criteria grafts. Therefore, significant effort has been dedicated to the development of improved cardiac allograft assessment techniques potentially capable of providing objective, quantitative, reproducible, and accurately predictive parameters. This review discusses briefly the currently available clinical tools for organ assessment (both in situ and ex vivo), before focusing on the novel experimental assessment techniques being developed by exploiting conventional imaging modalities capabilities (e.g., ultrasound, magnetic resonance and spectroscopy) that are being developed with the aim of achieving more objective and/or quantitative parameters for accurate, predictive ex vivo cardiac graft assessment.

## Current Assessment Techniques

Determination of an individual’s suitability to donate a cardiac allograft begins with an extensive review of the donor history. Favorable characteristics include no history of cardiac disease or related risk factors (family history of early-onset vasculopathy, hyperlipidemia, smoking, or obesity); age, with younger patients preferred, although donors over 60 have been used occasionally; cause of death; stable hemodynamics (mean arterial pressure > 60 mmHg, central venous pressure 8–12 mmHg); preserved end-organ function on no/low-level ionotropic support; absence of infectious diseases and/or high-risk donor behaviors with the potential to jeopardize the prospective recipient [4, 5]. Following this initial assessment of donor suitability, a given donor is matched to a given recipient based on AB0 compatibility, recipient wait list priority, gender, size, and weight matching. Once a donor is deemed appropriate for a recipient, hearts donated after brain death (DBD) and circulatory death (DCD) are commonly evaluated via echocardiogram, performed to assess ejection fraction, left ventricular wall thickness, regional wall motion, and valvular function. DBDs may be further evaluated via coronary angiograms if the donor age is over 40 and for younger patients with increased coronary artery disease risk and/or regional wall motion abnormalities on echocardiography.

After the pre-procurement graft assessment phase, the expected warm and cold ischemic time is the next critical parameter in determining the suitability of a donor for a given recipient. The vast majority of hearts recovered for transplantation (~ 97%) are DBD, which avoids any warm ischemic injury prior to cold storage, as the organs remain perfused with oxygenated blood until the point of procurement and initiation of ex situ preservation [6]. Alternatively, DCD hearts are inevitably exposed to warm ischemia during the time spent at body temperature after the blood supply has been reduced and aerobic metabolism halted. For metabolically active organs such as the heart, warm ischemia can lead to quick metabolic deterioration, with adverse hemodynamic consequences after reperfusion. In animal models and anecdotal clinical reports, duration of warm ischemia is proportionally associated with increased incidence of delayed cardiac graft function (PGD) and primary non-function (PGF). DCD hearts exposed to more than 30 min of warm ischemia time are generally considered untransplantable, although some transplant centers are already pushing this arbitrary boundary further [7]. Following organ procurement, DBD hearts are most commonly transported via static cold storage (SCS; ~ 4 °C) with newer temperature-controlled static cold preservation techniques (6–8 °C) extending the maximum planned cold ischemia time to 6 h as the outer limit of “acceptable” storage duration (e.g., Paragonix SherpaPak).

Recently, however, normothermic machine perfusion (NMP) has been introduced into the clinical setting as an alternative post-procurement organ handling technique with significant success (e.g., OSC). NMP solves many interrelated problems, including providing a platform to resuscitate and assess, both DCD and DDD organs, and potentially extend preservation duration, safely. NMP supports organs in a metabolically and mechanically active state that is conducive to metabolic and, potentially, functional assessment. NMP works by reestablishing oxygen/nutrient supply to the arrested heart via retrograde aortic perfusion, reviving the heart to allow metabolic recovery and assessment in an unloaded, non-working state. Once heart rhythm and contractility are restored, typically with ventricular pacing to a heart rate of 60–90 beats per minute, cardiac allografts are assessed subjectively by appearance (visual right ventricle distention and contractile function) with lactate levels and coronary flow being the only objective measurements of myocardial viability.

Clinically, assessment of cardiac grafts prior to transplantation is dependent on the organ handling/preservation technique (i.e., SCS vs. NMP). In the context of SCS, this assessment includes the re-consideration of all in situ parameters with age of the donor (IBM being a lesser, but important variable) and time under warm ischemia (< ~ 30 min for DCD only) and cold ischemia (< ~ 4 h for DBD only) being the most decisive parameters for transplantation. However, if these strict storage parameters are not met, no further assessment techniques are currently implemented to determine the relative viability of a given graft and it is usually deemed non-transplantable. Although, the static nature of this preservation modality (both in perfusate and metabolic activity) complicates the assessment via metabolic biomarkers, other techniques have the potential to assess and lessen the strictness of the viability parameters.

Similarly, assessment techniques for hypothermic machine perfused (HMP) hearts are very limited or nonexistent even though this perfusion method, in theory, may provide ample opportunity to measure parameters potentially predictive of graft viability and functional quality prior to transplantation. The opportunity to measure candidate metrics of organ viability arises via the access to the circulating perfusate and the theoretical ability to store the heart for longer periods due to the suppressed metabolic state and non-ischemic status of the organ [8]. The lack of progress in assessment techniques for HMP is likely due to the very sparse research being done in the realm of cardiac grafts in preclinical models, coupled with few published clinical trials reporting no graft assessment during ex vivo perfusion and prior to transplantation [9–11]. Although, over the years a couple clinical trials have reported the ability to store cardiac grafts via HMP for 18–24 h with positive outcomes and low incidence of PGD [10, 11].

In contrast, more options are available for graft assessment pre-transplantation for hearts preserved via NMP due to their active metabolic state. Recent research has demonstrated the capability to measure a wide array of functional parameters including vascular function, cardiac oxygen efficiency, levels of high-energy phosphates, and cardiac oxygen consumption in NMP grafts, although none have been implemented in the clinic [12, 13]. Similarly, a plethora of ‘conventional’ biomarkers, including candidate predictors of graft inflammation (interleukin 6—IL-6, vascular cell adhesion molecule 1—VCAM-1, P- and E-selectin, tumor necrosis factor‐α—TNF‐α), ischemic/reperfusion injury (TNF‐α), the expression of anti- coagulation factors (thrombomodulin), as well as myocyte injury/cardiomyocyte death (Troponin T), among others can be analyzed from the circulating perfusate [14–16]. As well as, less conventional biomarkers including microvesicle, messenger RNA and micro RNA phenotyping can be analyzed from cellular by- products circulating in the perfusate.

**Fig. 1:**
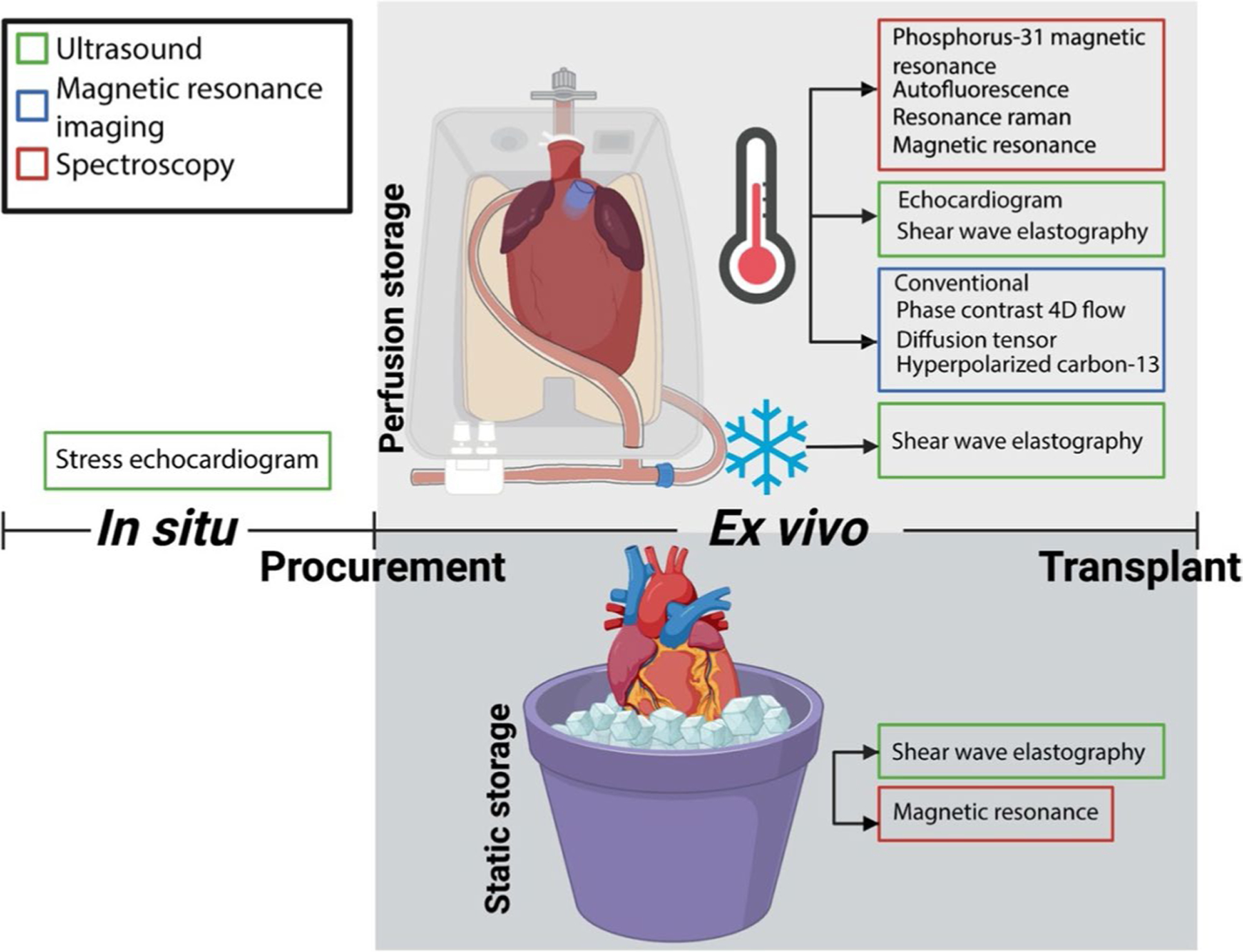
Novel imaging techniques usability for cardiac graft assessment based on of donor type (in situ vs. ex vivo) and storage modality (perfused normothermic vs. perfused hypothermic vs. static cold storage)

Despite the potential of biomarkers to determine relative graft viability, to date, neither experimental (previously mentioned) or clinically used (lactate) biomarkers have been shown to be predictive of cardiac graft function recovery or transplant success [17, 18]. Instead, many of the clinical and demographic criteria currently used to define transplantability of a particular heart into a particular recipient are applied subjectively and vary widely between surgeons and programs [19]. This subjectivity, coupled with the major adverse consequences associated with delayed graft failure of heart allografts, generally results in highly conservative utilization of cardiac grafts as a way to reduce recipient adverse events after transplantation. Therefore, extensive work is ongoing by the scientific community to standardize and simplify cardiac graft assessment as potential means to increase the number of hearts accepted for transplantation. In this capacity, this manuscript highlights the promise of imaging techniques, such as ultrasound, magnetic resonance imaging, and spectroscopy, in ex vivo heart assessment prior to decision for transplantation (Fig. 1).

## Novel Imaging-Based Experimental Assessment Techniques

### Ultrasound

Diagnostic ultrasound utilizes high frequency sound waves to obtain anatomical and functional information of the target organ. Current graft assessment via ultrasound has been limited to determining the viability of cardiac grafts in situ, as it determines the organ status by analyzing morphology (myocardial thickness, valvular structure) and functional status (global and regional contractility, valve performance). After hemodynamic stabilization and metabolic optimization, hearts with persistent global, or regional left ventricular dysfunction on a follow-up echocardiogram are often excluded from the donation process. Therefore, in an innovative attempt to increase the number of available grafts for transplantation, stress echocardiogram was utilized in a, pilot, clinical study to evaluate marginal hearts (determined by conventional assessment parameters) from DBD and resulted in the identification of grafts with severe morphologic abnormalities, as well as grafts with transplantable potential [20]. The marginal hearts with transplantable potential by the stress echo underwent improvement in regional wall motion during the test which translated to normal function and coronary perfusion after transplantation [21]. Through this assessment method, some additional grafts deemed marginal by conventional echocardiogram assessment can be considered for transplantation. If more broadly applied, this approach could potentially lead to an increase in available hearts for transplantation. However, it should be noted that hemodynamic management of DBD organ requires pharmacological interventions that might have adverse consequences for other organs or precipitate cardiac arrhythmias, potentially jeopardizing the organ recovery process. Furthermore, these “heart challenge” approaches are not feasible for DCD hearts assessment due to the ethical considerations of applying non-therapeutic treatments in living patients.

Due to the clinical importance to assess cardiac grafts during preservation, different approaches have been taken to enable echocardiography during NMP heart preservation. For instance, the incorporation of echocardiogram measurements into left ventricular function assessment in porcine and human grafts preserved via NMP machine perfusion is relatively novel, and only currently being performed in the experimental setting [22, 23]. Although a standard assessment technique, echo has not been widely implemented for the assessment of machine perfused grafts due to the lack of heart chamber loading and physiological blood pumping of grafts perfused in non-working Langendorff mode (the current clinical standard on the OCS). To circumvent these issues, experimentally, the left heart chambers of porcine and human hearts were filled (eliminating air), which reestablished physiological loading conditions, either, on the left side only or in both sides of the heart [22, 24]. To achieve this, perfusate was flowed through the superior vena cava and/or through the pulmonary vein into the atriums of the heart, instead of the aortic root, and allowed to be circulated into the corresponding ventricles by the contractile function of the heart and out of the heart against a resistance, which mimics systemic vascular resistance, although inaccurately due to the lack of compliance. This method of heart perfusion is commonly known as working heart mode, which due to significantly greater logistical challenges (accurate pre-load, after-load, synchronous pumping, etc.), is rarely utilized during clinical machine perfusion preservation drastically reducing the practicality of this experimental method. Furthermore, ventricular volume and pressure loading increases metabolic demands, stressing the myocardium metabolically and mechanically, increasing the risk of injury, possible exacerbating injury by warm ischemia and/or ischemia reperfusion injury.

Fortunately, other ultrasound techniques have demonstrated higher potential to evaluate cardiac grafts ex vivo, without the need to modify the preservation technique. For instance, cardiac tissue stiffness, measured via shear wave velocity, has been introduced as a potential biomarker for graft health. Shear wave velocity (SWV) is an ultrasound technique capable of determining changes in tissue stiffness by calculating the velocities in which sound waves travel on the surfaces of the heart. It is considered to be independent of heart loading conditions which enable its implementation before storage and during different preservation modalities. This assessment technique consists of quantifying changes in myocardial stiffness as a function of preservation time under SCS (cold ischemia) and harvesting conditions (warm ischemia), where stiffness was determined to increase proportional to, both, cold and warm ischemia durations in porcine cardiac grafts [25]. Therefore, a high correlation between SWV values and cardiac function parameters such as contraction efficiency and relaxation rate were determined, as well as a moderate correlation between SWV values and graft workload and end-diastolic pressure [25]. SWV has also been utilized to perform similar measurements on rabbit hearts during Langendorff machine perfusion and working heart modality, where the SWV values correlated to myocardial contractility, diastolic function, and intraventricular pressure [26–30]. The value of SWV as an assessment technique, if further developed, could be immense due to its capabilities for providing quantitative measurements of a given graft’s relative transplantability preserved via SCS or machine perfusion. Hence, the quantitative nature of this assessment technique has great potential to eliminate decision subjectivity (Table 1).

The applicability of an ultrasound- based assessment technique in the clinical setting seems extremely likely due to the benign nature of the technique. Ultrasound is a well-developed and rapidly advancing imaging method, widely used in clinical diagnostics. Furthermore, the imaging system generates no radiation and is highly portable, with ultrasonic probes connecting to pocket phones, facilitating the implementation of the technology. The current biggest gain of implementing ultrasound as an assessment technique comes from the ability to assess cardiac grafts stored in, both, cold and normothermic temperatures, a feature other assessment techniques do not possess, making ultrasound and SWV extremely relevant to cardiac graft assessment prior to transplantation.

### Magnetic Resonance Imaging

Magnetic resonance imaging (MRI) utilizes strong magnetic fields to polarize hydrogen atoms within the tissue to generate a radio frequency signal used to create images of organs within the body [31]. Since hydrogen atoms are naturally abundant in water and fat, MRI produces superior soft tissue contrast capable of functional, anatomical, and metabolic imaging [32]. Conventional MRI has been utilized experimentally to measure wall motion and intramyocardial strain in human hearts revived using a four-chamber working perfusion system adapted to be MRI compatible [33]. The strength of MRI as an assessment technique lies on the versatility of its imaging capabilities. For instance, via the utilization of gadolinium-DPTA, a biocompatible contrast medium commonly used to image blood vessels, MRI has been utilized to determine the adequacy of graft perfusion [34]. Additional to determining lack/presence of flow through the coronaries, MRI has also been utilized to measure blood flow velocities within the loaded heart chambers of the working heart via phase contrast technique [35]. Phase contrast MRI, also known as 4D flow MRI, facilitates the accurate visualization of three spatial dimensions, plus time, which provide valuable information regarding intracardiac blood flow, vortex detection, and assessment of flow through the heart valves [36–38]. To date only initial feasibility studies in a porcine working heart model have been performed on the relative usability of 4D flow MRI to image grafts within a machine perfusion setup, which demonstrated the capability to acquire accurate flow visualizations [39]. Although the information that can be obtained from 4D flow MRI would be extremely valuable, this technique is highly impractical at the time of determining the potential usability of cardiac grafts for transplantation. First, its usefulness is confined to evaluating the perfused heart in working mode, a significant challenge for NMP as discussed above. Second, 4D flow MRI depends on sophisticated, bulky, non-portable equipment, can only be performed in a magnetically shielded environment, and requires extensive imagining time and advance image processing software, making this technique impractical for clinical use [40].

Another MRI functional imaging capability extremely relevant to cardiac graft assessment is diffusion tensor MRI, which utilizes specific signal sequences and specialized software to map the diffusion process of molecules within biological tissues. Via the parameters derived from this technique (fractional anisotropy — FA), direct measurement of endothelial and cardiomyocyte leaky-ness and viability can be obtained. In a canine beating heart model, strong correlation between FA values and parameters of functional recovery (developed pressure, rate of pressure generation, and rate of relaxation) have been reported [18]. Although FA values also correlated with tissue levels of conventional biopsy-based biochemical viability markers (ATP-levels, endothelin-1, malondialdehyde, and caspase-3), recovery of myocardial function did not correlate with modulation of these markers or any other conventional analyses of myocardial injury [18]. The lack of correlation between commonly used viability markers and myocardial function strongly supports the need for the development of improved and accurate assessment techniques.

Via the utilization of this technique, important information regarding the functionality of the vasculature and myocardium at the cellular level can be obtained via a rapid, non-invasive scan.

A third MR technique with the potential to aid in the assessment of cardiac grafts during machine perfusion is hyperpolarized carbon-13 MRI. This modality of MRI consists on hyperpolarizing molecules that contain carbon-13 via dynamic nuclear polarization and rapid dissolution to create a metabolizable solution [41]. This hyperpolarized substrate is added to the perfusate, metabolized by the organ over time, and traced chemically and spatially via MR imaging. For cardiac graft assessment [1-^13^C] pyruvate has recently been tested as tracer due to its central role in cellular metabolism as it leads to energy production and the formation of lactate, alanine, and carbon dioxide [32, 42]. The resulting metabolic profile correlated with aspects of Langendorff machine perfused porcine hearts were the pyruvate signal was only localized in the myocardium and not in the chambers of the heart [32]. Furthermore, differences in the metabolic profile of machine perfused heart and in vivo measurement were significant and correlated with differences in functional measurements, which indicates good sensitivity of this MRI method [32]. The capability to measure changes in metabolic activity non-invasively is extremely valuable due to the ability to associate these changes with myocardial functional decline, without the need for biopsies or time-consuming metabolomics analysis [43]. It is important to point out that some degree of differences between in vivo and perfused metrics are expected due to the differences in overall heart perfusion and workload, and do not necessarily indicate damaged/non-transplantable grafts. Instead a metabolic profile baseline for transplantable grafts can be obtained and tested experimentally to determine its usability to assess the relative transplantability of machine perfused hearts (Table 2).

Although MR is a powerful imaging technique with high potential to improve cardiac graft assessment, it currently possesses two draw-backs that have limited the implementation of these techniques in the clinical setting. First, MRI as an assessment tool can only be used for machine perfused graft as metabolic and functional parameters are not measurable on SCS grafts. Second, and likely more important, the magnetic nature of this technique requires the existing machine perfusion systems to be reconfigured to contain no magnetizable metals. Unless there is a major demonstrated advantage for MR over other approaches, there is no scientific or commercial impetus for the field to re-build a system that has already been FDA approved and works relatively well. However, the capability of MRI to provide a wide array of assessment parameters (edema, fibrosis, perfusion, and cellular health, as well as cardiac output, end diastolic volume, ejection fraction, end systolic volume, heart rate, and stroke volume) generally acquired with multiple techniques (biopsies, perfusate analysis, etc.) within a single imaging scan could significantly simplify cardiac graft assessment and might justify the necessary changes to the current perfusion systems [32, 44–46]. However, it is likely that MRI and related MRI-dependent technologies will only be utilized in the experimental setting for the foreseeable future.

### Spectroscopic Techniques

Spectroscopy measures and interprets the wavelengths or frequencies resulting from the absorption, emission, or reflection of light and other radiation by matter. In biomedical applications, spectroscopy is utilized to determine the chemical composition of tissues and rapidly quantify molecular components based of the vibrational modes of its constituent molecules. Different types of spectroscopic approaches utilize different excitation mediums which result in a wide array of applications. For instance, magnetic resonance spectroscopy (MRS), also known as nuclear magnetic resonance spectroscopy, utilizes a strong, constant magnetic field to align the molecules’ nuclei, followed by the perturbation of this alignment by a weak oscillating magnetic field [47]. This perturbation causes precession of the nuclear spins and, in turn, induces characteristic voltages, which can be measured and analyzed. MRS scans can be tuned to pick up signals from different chemical nuclei within the body, being capable of measuring a wide array of biochemical compounds. For instance, Phosphorus-31 magnetic resonance (^31^P MRS) has been utilized to non-invasively measure the intracellular levels of phosphorus metabolites in porcine cardiac grafts during Langendorff machine perfusion [48]. As a result, inorganic phosphate, phosphocreatine (used for maintenance and recycling of ATP) and the α-, β-, and γ- phosphate moieties of ATP were successfully tracked and quantified over time, providing significant dynamic insight into the energetic state of cardiomyocytes. The resulting energetic profiles were utilized to successfully distinguish grafts with experimentally induced ischemia from non-ischemic ones [48]. MRS has also been used to measure lactate, creatine, and phosphocreatine from porcine cardiac grafts preserved via SCS and hypothermic machine perfusion [49]. Similar to ^31^P MRS, the energetic profile obtained correlated to the degree of functional recovery for grafts in both preservation modalities, where the hearts preserved via hypothermic machine perfusion demonstrated improved energetic and graft function relative to those statically stored. Interestingly, the measurements in the preserved grafts were obtained from biopsies and not from the entire organ as it was done in the ^31^P MRS study; adding an important versatility to MRS analysis. Although, the use of biopsies would eliminate the non-invasive characteristic of MR assessment, it would also solve the need to re-build a non-magnetizable perfusion system. This technique has immense potential in the field of cardiac transplantation, as being able to determine and quantify ischemia damage and graft function after preservation is crucial in determining graft suitability for transplantation.

Another spectroscopy technique utilized for the assessment of cardiac grafts is autofluorescence (AF) spectroscopy, also known as native fluorescence. AF consists on measuring the natural emission of light from biological structures resulting from the excitation with light at molecule-specific wavelengths [50]. Two crucial molecules in the mitochondrial electron transport chain, nicotinamide adenine dinucleotide (NADH), and flavin adenine dinucleotide (FAD), change the intensity of their autofluorescence depending on their relative redox state; serving as surrogates to determine myocardial metabolic function [51, 52]. This technique has been utilized to measure the redox state of NADH and FAD in rat and rabbit cardiac grafts under Langendorff machine perfusion, subjected to regional or global ischemia, as well as varying degrees energetic demands [52, 53]. Importantly, these studies, provided evidence that the levels of NADH/FAD reliably changed as a function of ischemia, reperfusion, and energetic demands, where NADH (increased) and FAD (decreased) levels changed as expected, as a function of increased ischemia time and the levels of NADH increased as metabolic demand was increased [52, 53]. Although both metabolic fluorophores were successfully utilized as indicators of cardiac metabolism in the research setting, in the clinical setting only NADH would be measurable due to the significant interference of hemoglobin with FAD fluorescence [52]. Regardless, the sensitivity of this relatively simple technique to assess metabolic changes via the measurement of NADH alone has great potential for simplifying and standardizing experimental and clinical cardiac graft assessment.

Resonance Raman spectroscopy (RRS) is one of the newest spectroscopy techniques being developed for the assessment of grafts viability for transplantation. RRS utilizes monochromatic light (visible, near infrared, or near ultra-violet) to interact with the vibrational modes of molecules to inelastically scatter light and generate a unique spectrum proper of each molecule. Libraries of molecules are created with spectral markers generated from the analysis of purified compounds, which are later utilized to identify the molecules in complex tissue measurements. For cardiac graft assessment, spectral libraries for myoglobin, hemoglobin, and mitochondrial cytochromes in both the oxidized and reduced states were created from isolated compounds [54]. The information obtained from the mitochondrial cytochromes redox state was utilized to compute the ratio of reduced to total mitochondria (Raman reduced mitochondrial ratio — 3RMR), which was then used as a potential metric of cardiac health. The sensitivity and specificity of 3RMR as an indicator of myocardial energy production was determined to successfully reflect episodes of, both, ischemia and hypoxemia with changes in 3RMR reliably predicting the reduction in cardiac contractility in rats [54]. Consistently, episodes of decreased cardiac contractility were followed by increases in myocardial 3RMR, suggesting deficiencies in electron transport process [54]. This technique represents a novel method to assess the adequacies of oxygen delivery to cardiac grafts, while providing critical insight into the effective redox states of mitochondria. Alternatively, this is only one of the many diagnostic applications that could be developed with RRS technologies. The wide array of molecules with resonance capabilities within biological tissue, and the ability to modify the wavelength of the emitted light to meet specific needs give RRS an important diagnostic flexibility, currently not available in other candidate assessment modalities (e.g., the capability to also measure Cytochrome C). Furthermore, the high specificity, real-time, non-contact capabilities of RRS, plus the portability of the system allow easy integration with current heart preservation systems. An important limitation of RRS is the inability to assess cardiac grafts preserved by cold storage (SCS and HMP), as the reported 3RMR number from well-preserved, presumed viable grafts tends to be statistically the same as non-transplantable ones [55]. This may be due to the fact that both grafts are exposed to the same degree of ischemia during storage and the viability difference is determined by the ability of the graft to normalize its energetic profile and recover after the preservation period has ended. Although, it has not been reported, this limitation may also apply to the measurement of NADH and FAD from cold stored grafts (Table 3).

The feasibility of the utilization of any of the spectroscopy techniques seems variable. Although magnetic resonance spectroscopy is widely used in the clinical setting as a diagnostic tool, the implementation of these techniques for the assessment of cardiac allografts faces the same challenges as all other MR-based techniques. On the other hand, although all spectrophotometry techniques can easily be implemented for cardiac graft assessment utilizing simpler, portable system, they have yet to be widely implemented or adopted in the clinical setting. This implies these techniques have a long road of regulatory and safety development before they can be used regularly in the clinical setting.

## Conclusion

The ability to accurately assess graft quality in situ or during storage before implantation is fundamentally to permit the safe utilization of hearts that currently are not utilized. A wide array of unmodifiable clinical factors, including expected cold ischemic time for DCD hearts and variable warm ischemia time for DCD hearts (loss of energy and cellular homeostasis vs. the ability to recover), donor age, etc., correlate with the probability of transplant success based on registry data. Absence of a technique to accurately predict the ability of a heart to recover that has been deemed ‘marginal’ based on one or more of these risk factors leads to a conservative decision-making that prevents clinical utilization of the majority of cardiac grafts from global donors [56]. Therefore, an improved ability to assess donor cardiac function using a combination of imaging and biometric parameters has the promise to expand the numbers of hearts used for transplantation, especially from DCD and older donors. As a result, further and significant effort should be focused on the development of improved assessment techniques capable of dynamically, objectively and quantitatively determining the relative viability of cardiac grafts. In our estimation, combining imaging technologies with established and emerging biochemical and metabolic assessments is an attractive approach to overcome the limitations associated with cardiac graft assessment technologies, due to their undisputed success as diagnostic tools in other fields of medicine.

## Figures and Tables

**Table 1:** Assessment capabilities of ultrasound techniques to determine cardiac allograft relative viability

Ultrasound technique	Assessment parameters	Clinical/experimental setting	Type of donor	Mode of preservation
	Myocardial thickness Valvular structure			
Stress echocardiogram	Global and regional contractility	Clinical trial	In situ DBD	N/A
	Valve performance Regional wall motion			
Echocardiogram	Right and/or left ventricular function	Experimental	Ex vivo DBD or DCD	Perfused in working heart mode only
Shear wave elastography	Cardiac tissue stiffness			Perfused SCS

**Table 2:** Assessment capabilities of magnetic resonance imaging (MRI) techniques to determine cardiac allograft relative viability. MRI assessment techniques have been experimentally utilized to acquire a wide array of functional and metabolic markers from DBD and DCD cardiac grafts perfused ex vivo

Magnetic resonance imaging technique	Contrast medium	Assessment parameters
Conventional MRI	N/A	Wall motion intramyocardial strain
	Gadolinium-DPTA	Adequate graft perfusion Intracardiac blood flow
Phase contrast MRI 4D flow	N/A	Vortex detection Flow through heart valves
Diffusion tensor MRI	N/A	Endothelial/cardio myocyte leaky-ness and viability
Hyperpolarized carbon-13 MRI	[1–13C] pyruvate	Cellular energetics (lactate, alanine, carbon dioxide)

**Table 3:** Assessment capabilities of spectroscopy techniques to determine cardiac allograft relative viability. Spectroscopy techniques have been experimentally utilized to acquire a wide range of cellular energetic markers from DBD and DCD cardiac grafts perfused ex vivo

Spectroscopy technique	Assessment parameters	Mode of preservation
Phosphorus-31 magnetic resonance spectroscopy	Inorganic phosphate, phosphocreatine and the α-, β-, and γ-phosphate moieties of ATP	
Autofluorescence spectroscopy resonance Raman spectroscopy	Redox state of NADH and FAD redox state of myoglobin, hemoglobin, and mitochondrial cytochromes	NMP
Magnetic resonance spectroscopy	Lactate, creatine, and phosphocreatine	NMP SCS
